# Galectin‐1 targets brain endothelial cell changes, reduces vascular amyloid deposition and restores blood‐brain barrier (BBB) integrity in Alzheimer's Disease models

**DOI:** 10.1002/alz70855_107216

**Published:** 2025-12-24

**Authors:** Jessica L Presa, Carlos Javier Pomilio, Eugenia Matzkin, Agustina Alaimo, Mariano Soiza Reilly, Montana Manselle, Joaquin Merlo, Soledad Gori, Rossana Ramhorst, Juan Beauquis, Gabriel A Rabinovich, Flavia Saravia

**Affiliations:** ^1^ University of Buenos Aires, Buenos Aires, Argentina; ^2^ CONICET, Buenos Aires, Buenos Aires, Argentina; ^3^ CONICET, Buenos Aires, Argentina; ^4^ IQUIBICEN ‐ CONICET, Ciudad Autónoma de Buenos Aires, Buenos Aires, Argentina; ^5^ IFYBIME‐ CONICET, Ciudad Autónoma de Buenos Aires, Buenos Aires, Argentina; ^6^ CONICET, Ciudad Autónoma de Buenos Aires, Buenos Aires, Argentina; ^7^ CONICET, Ciudad Autónoma de Buenos Aires, Argentina; ^8^ University of Buenos Aires, Ciudad Autónoma de Buenos Aires, Buenos Aires, Argentina

## Abstract

**Background:**

Alzheimer's disease (AD) is characterized by neuroinflammation, vascular dysfunction, and impaired proteostasis. Galectin‐1 (Gal1), a carbohydrate‐binding protein with immunomodulatory and vascular regulatory roles, emerges as a potential therapy for AD.

**Methods:**

We integrated in vitro and in vivo AD experimental models to evaluate the role of Gal1 in AD pathophysiology using human brain microvascular endothelial cells (HBMECs) exposed to Aβ1‐40 and PDAPPJ20 transgenic (Tg) mice treated with Gal1 (9 doses of 100 µg). We evaluated the impact of Gal1 administration on AD‐related changes using multiple approaches including immunofluorescence, confocal microscopy, flow cytometry, transwell, RT‐PCR, BBB permeability assays, and RNAseq. Custom macros in FIJI were used for image analysis. Parametric and non‐parametric statistical analyses, where appropriate, were performed using R/Graphpad software.

**Results:**

In vitro, Aβ1‐40‐exposed HBMECs upregulated endogenous Gal1 protein at subtoxic concentrations, suggesting a compensatory protective mechanism (*p* <0.05). Gal1 directly bound HBMECs via its carbohydrate recognition domain and prevented Aβ‐induced BBB disruption in a transwell model (*p* <0.05). In addition, Gal1 restored Aβ 1‐42 uptake by HBMECs (*p* <0.05), probably through improved proteostasis and consequently intracellular trafficking. Accordingly, Gal1 counteracted Aβ‐induced prolonged activation of the unfolded protein response effectors BIP(*p* <0.01), IRE1, PERK, and XBP1 (all *p* <0.05), as well as the NLRP3 inflammasome in HBMECs (*p* <0.05).

In vivo, Gal‐treated Tg mice showed a significant reduction in perivascular amyloid deposition in the hippocampus (*p* <0.05). Astrocyte‐endothelial interactions, critical for blood‐brain barrier (BBB) integrity, were restored as evidenced by increased astrocytic endfeet contact (GFAP‐lectin staining) and perivascular aquaporin‐4 colocalization (*p* <0.02). BBB permeability was reduced in Gal1‐treated Tg mice (Evans blue assay, *p* <0.05). RNAseq analysis of whole hippocampal brain tissue from Tg mice revealed extensive alterations in key pathways related to immune function, neuronal activity and proteostasis networks, while Tg‐Gal1 mice showed a restored transcriptional profile similar to control mice. Gal1‐treated Tg mice showed reduced XBP1 vascular presence in the hippocampal hilus compared to untreated mice (*p* <0.05), further validating the in vitro results.

**Conclusions:**

These novel findings position Gal1 as a pleiotropic modulator of AD pathology, with a focus on microvascular changes. Given the multifactorial nature of this pathology, Gal1‐based strategies could target disease mechanisms at multiple levels.

